# Radiofrequency technology as an anti-aging intervention: clinical efficacy in reversing senescence-driven skin laxity and age-related dermal degeneration

**DOI:** 10.3389/fcell.2026.1866750

**Published:** 2026-07-20

**Authors:** Muyang Wang, Min Fu, Jiayi Zhang

**Affiliations:** 1 Medical Aesthetics Center, The People’s Hospital of Longhua, Shenzhen, Guangdong, China; 2 Department of Dermatology, The People’s Hospital of Longhua, Shenzhen, Guangdong, China

**Keywords:** age-related elasticity decline, aging skin, anti-aging aesthetics, cellular senescence, clinical efficacy, monopolar radiofrequency, multipolar radiofrequency, senescence-associated dermal degeneration

## Abstract

**Objective:**

To compare the clinical anti-aging efficacy of multipolar radiofrequency technology and monopolar radiofrequency technology in reversing senescence-driven skin laxity and age-related dermal degeneration, and to provide scientific evidence for the selection of optimal radiofrequency anti-aging treatment protocols in aging patients.

**Methods:**

A retrospective analysis was conducted on the clinical data of 84 aging patients exhibiting senescence-associated skin laxity and age-related dermal extracellular matrix degeneration, who received radiofrequency anti-aging treatment at our hospital from January 2022 to January 2024. Patients were divided into a study group (multipolar radiofrequency treatment, n = 42) and a control group (monopolar radiofrequency treatment, n = 42) according to the treatment method. Both groups received 5 treatments, once every 2–3 weeks. Skin elasticity parameters (R2, R5, R7) — reflecting collagen and elastin fiber degradation—were measured using a cutometric device, aesthetic improvement was evaluated using GAIS, skin texture was assessed using a three-dimensional skin analyzer, and patient satisfaction was evaluated using VAS and PSES.

**Results:**

Senescence-associated skin elasticity parameters reflecting age-related dermal structural decline improved significantly in both groups at all follow-up time points compared to aging pre-treatment baseline (P < 0.05), confirming that both radiofrequency modalities can partially reverse senescence-driven dermal deterioration. At 3 months post-treatment, the improvement rates of aging biomarker parameters R2, R5, and R7 in the study group were 32.7% ± 5.2%, 28.9% ± 4.6%, and 30.8% ± 5.1%, respectively—representing substantially greater reversal of age-related elasticity decline—compared to 21.5% ± 4.1%, 18.7% ± 3.9%, and 20.1% ± 4.0% in the control group (P < 0.001). Anti-aging GAIS scores were higher in the study group at all follow-up time points (P < 0.001). Age-related skin texture deterioration showed superior improvement in the study group across senescence-associated skin roughness index, age-enlarged pore area and density, aging fine line depth, and texture uniformity (P < 0.001). VAS and PSES scores reflecting patient-perceived aging reversal were higher in the study group at all time points (P < 0.001). At 12-month follow-up, 90.5% of study group patients maintained effective anti-aging improvement versus 57.1% in the control group (P < 0.001), demonstrating superior durability of senescence-reversal. No significant difference in adverse reaction incidence between groups (P > 0.05).

**Conclusion:**

Multipolar radiofrequency technology is superior to monopolar radiofrequency technology in reversing skin laxity and dermal degeneration associated with aging, demonstrating greater improvements in skin elasticity parameters, overall aesthetic outcomes, patient satisfaction, and treatment durability, with a comparable safety profile, making it the preferred option for non-invasive skin tightening in older patients.

## 1 Introduction

With the acceleration of global population aging, skin aging—driven by the progressive accumulation of senescent cells, age-related extracellular matrix remodeling, and chronic low-grade sterile inflammation known as inflammaging—has become one of the most clinically significant dermatological challenges of our era. At the cellular level, aging dermal fibroblasts undergo replicative senescence characterized by telomere attrition, upregulation of cyclin-dependent kinase inhibitors (p16INK4a, p21), and adoption of the senescence-associated secretory phenotype (SASP), which drives paracrine secretion of matrix metalloproteinases (MMPs) that degrade collagen and elastin scaffolds. The resulting senescence-driven skin laxity, decreased elasticity, and deepening of fine lines not only alter individual appearance but also carry profound negative impacts on psychological health and quality of life (Flinn et al., 2025). Traditional skin tightening treatment methods include surgical facelifts and injection fillers, but these approaches carry significant trauma, prolonged recovery periods, and high risk of complications poorly tolerated by aging patients with diminished tissue repair capacity (Clark et al., 2023). Therefore, seeking safe and effective non-invasive anti-aging treatment technologies capable of stimulating senescence-impaired fibroblasts to restore collagen production has become an important research direction in the field of medical aesthetics.

Radiofrequency (RF) technology, as an emerging non-invasive anti-aging treatment modality, directly targets the biological consequences of skin aging by delivering controlled thermal energy to deep dermal layers. This controlled thermal stimulus activates heat-shock protein pathways in senescent fibroblasts, partially overcoming the age-related transcriptional silencing of collagen synthesis genes (COL1A1, COL3A1), stimulating collagen denaturation, contraction, and neo-collagenesis, and thereby partially reversing the age-driven structural deterioration of the dermal extracellular matrix (Bai et al., 2025). In recent years, RF technology has been widely applied in aging skin restoration, and its anti-senescence safety and efficacy have gradually gained recognition (Shu et al., 2022; Wang et al., 2025). According to different electrode configurations, RF technology is divided into two types: monopolar RF and multipolar RF. Monopolar RF penetrates deeply into aging dermal tissue but its energy distribution is relatively uneven, creating thermally heterogeneous stimulation of aging fibroblast populations (Wanitphakdeedecha et al., 2022). Multipolar RF achieves more precise energy control and uniform heat distribution through energy transfer between multiple electrodes, theoretically providing more homogeneous thermal stimulation across the treated tissue volume and potentially delivering superior therapeutic effects in skin laxity applications (Wattanakrai et al., 2022).

Although the application of RF technology in aging skin restoration has become increasingly mature, comparative studies evaluating which RF modality more effectively reverses senescence-driven dermal degeneration remain limited. Particularly for the anti-aging efficacy comparison between multipolar RF and monopolar RF in treating age-related skin laxity, there is a lack of large-sample, long-term follow-up systematic studies that account for the biological heterogeneity of aging skin (Khong et al., 2024). Therefore, this study retrospectively analyzes and compares the clinical anti-aging efficacy of multipolar RF technology and monopolar RF technology in senescence-driven skin laxity treatment, objectively evaluating the differences between the two technologies in reversing age-related skin elasticity decline, anti-aging aesthetic effects, patient satisfaction with aging reversal outcomes, and durability of senescence-countering treatment effects, to provide scientific evidence for clinical selection of the most appropriate RF anti-aging protocols and promote the standardized application of RF technology in age-related skin restoration.

## 2 Materials and methods

### 2.1 Study design and subjects

This study retrospectively analyzed the clinical data of aging patients exhibiting senescence-driven skin laxity and age-related dermal structural deterioration who received radiofrequency anti-aging treatment at our hospital from January 2022 to January 2024. The study protocol was approved by the hospital ethics committee. According to the treatment method received, 84 patients meeting inclusion criteria were divided into two groups: the study group (n = 42) received multipolar RF anti-aging treatment, and the control group (n = 42) received monopolar RF anti-aging treatment.

#### 2.1.1 Inclusion criteria

(1) Age 25–65 years, spanning the range from early through advanced biological skin aging, regardless of gender; (2) Patients presenting with clinically evident senescence-associated facial skin laxity and age-related elasticity decline requiring anti-aging restoration; (3) Complete clinical data with 12-month follow-up completion; (4) No previous anti-aging skin tightening treatments before enrollment; (5) No severe systemic diseases that would impair aging-tissue response to thermal stimulation.

#### 2.1.2 Exclusion criteria

(1) Pregnant or lactating women; (2) Active skin infection, malignant tumors, or precancerous lesions in the treatment area; (3) Keloid constitution or pathological wound healing reflecting aberrant aging-related tissue repair; (4) Facial surgery or other cosmetic anti-aging treatments within the past 6 months; (5) Severe heart, liver, or kidney dysfunction impairing systemic tolerance to thermal anti-aging procedures; (6) Mental illness or cognitive disorders affecting treatment cooperation; (7) Allergy to treatment device materials; (8) Incomplete clinical data or lost to follow-up patients.

Both groups of aging patients showed no statistically significant differences in chronological age, biological skin aging severity, gender, BMI, skin phototype, previous cosmetic anti-aging treatment history, baseline senescence-associated skin condition, comorbidity burden, and medication history (P > 0.05), ensuring comparable aging biological context between groups. Detailed baseline demographic and clinical characteristics for both groups are presented in Table 1. Subsequent data tables are renumbered accordingly (formerly Table 1 through Table 7 are now designated Table 2 through Table 8).

**TABLE 1:** Baseline characteristics of both groups.

Characteristic	Study group (n = 42)	Control group (n = 42)
Age (years, x̄±s)	43.8 ± 9.4	44.5 ± 8.7
Female, n (%)	35 (83.3%)	36 (85.7%)
BMI (kg/m^2^, x̄±s)	22.6 ± 2.8	22.9 ± 3.1
Fitzpatrick phototype III/IV, n (%)	38 (90.5%)	39 (92.9%)
Baseline R2 (%)	0.62 ± 0.08	0.61 ± 0.09
Prior cosmetic treatment, n (%)	0 (0%)	0 (0%)

All between-group comparisons P > 0.05 (independent samples t-test or chi-square test, as appropriate). Baseline skin laxity severity, comorbidity burden, and medication history also showed no significant between-group differences (data not shown).

**TABLE 2:** Comparison of skin elasticity parameter improvement between two groups.

Parameter	Group	1 Month post-treatment	3 Months post-treatment	6 Months post-treatment	12 Months post-treatment
R2 improvement rate (%)	Study group	28.4 ± 4.8	32.7 ± 5.2	29.8 ± 4.9	24.6 ± 4.1
	Control group	18.2 ± 3.9	21.5 ± 4.1	19.3 ± 3.8	15.7 ± 3.2
	t value	10.432	10.847	10.278	11.053
	P value*	<0.001	<0.001	<0.001	<0.001
R5 improvement rate (%)	Study group	24.8 ± 4.2	28.9 ± 4.6	26.3 ± 4.4	21.2 ± 3.8
	Control group	16.1 ± 3.5	18.7 ± 3.9	17.2 ± 3.6	13.8 ± 2.9
	t value	9.756	10.214	9.643	9.987
	P value*	<0.001	<0.001	<0.001	<0.001
R7 improvement rate (%)	Study group	26.5 ± 4.5	30.8 ± 5.1	28.1 ± 4.7	23.4 ± 4.0
	Control group	17.3 ± 3.7	20.1 ± 4.0	18.5 ± 3.7	14.9 ± 3.1
	t value	9.873	10.568	9.892	10.341
	P value*	<0.001	<0.001	<0.001	<0.001

P values corrected by Bonferroni method.

### 2.2 Treatment methods

The study group received multipolar radiofrequency anti-aging treatment targeting senescence-impaired dermal fibroblasts and age-degraded extracellular matrix, using the following parameters: ① Frequency 4–6 MHz optimized for penetration to aging dermal depths; ② Power 10–50W, individually adjusted according to aging skin tolerance and thermal sensitivity; ③ Multipolar radiofrequency probe with contact area 2–4 cm^2^; ④ Treatment protocol: once every 2–3 weeks, 5 treatments total, 30–45 min per session, timed to allow age-impaired tissue recovery between sessions; ⑤ Stepwise incremental protocol: sessions 1–2 at 20–30W (allowing acclimatization of aging tissue), sessions 3–4 at 30–40W, session 5 at 40–50W, with skin surface temperature controlled within 42 °C–52 °C—the therapeutic range proven to activate senescence-impaired collagen synthesis pathways without inducing thermal senescence of additional fibroblasts.

The control group received monopolar radiofrequency anti-aging treatment (frequency 4–6 MHz) with power 15–40W individually adjusted for aging skin tolerance, treatment interval 2–3 weeks, 5 treatments total, 30–45 min per session, with skin surface temperature maintained within 40 °C–50 °C. All aging patients underwent detailed medical history collection—including assessment of chronological age, aging-related comorbidities, and previous senescence-targeted treatments—physical examination, treatment area cleaning and disinfection, standardized clinical photography documenting baseline aging appearance, and informed consent signing before treatment. Post-treatment care instructions were uniformly provided for aging skin: avoid hot water face washing for 24 h to protect thermally stimulated aging tissue, use medical cold compress masks to calm senescence-associated post-treatment inflammation, strict sun protection (SPF≥30) to prevent photoaging compounding intrinsic aging, use gentle moisturizing products to support compromised aging skin barrier function, and avoid irritating cosmetics that may exacerbate inflammaging.

The difference in surface temperature ceilings between the two groups (42 °C–52 °C for multipolar RF versus 40 °C–50 °C for monopolar RF) reflects a deliberate and clinically reasoned protocol rather than an inconsistency. The multipolar RF system distributes radiofrequency energy across a larger tissue contact area through simultaneous multi-electrode conduction, resulting in lower energy density per unit tissue area at equivalent total power output. Achieving equivalent therapeutic heating depth in aging dermal tissue therefore requires modestly higher total power input, which translates to a slightly higher monitored surface temperature ceiling without exceeding the safe thermal threshold for aging skin. The 2 °C differential was established empirically through device calibration and validated against real-time infrared thermometry during pilot cases prior to study commencement, consistent with the manufacturer’s published clinical safety parameters for this device configuration. In both groups, real-time surface temperature monitoring was performed throughout each treatment session, with immediate power reduction triggered if readings approached the ceiling, ensuring that thermal exposure in both groups remained within clinically established safe ranges.

### 2.3 Observation indicators

#### 2.3.1 Primary observation indicators

##### 2.3.1.1 Skin tightening improvement rate

Senescence-associated skin elasticity parameters of bilateral cheek areas—the facial region most susceptible to age-related gravitational laxity and collagen loss—were measured using a skin elasticity measuring device (Cutometer MPA 580, Courage + Khazaka, Germany). These parameters are direct biomechanical surrogates of age-related extracellular matrix integrity: ① Immediate elastic recovery rate (R2, reflecting aging elastic fiber function and age-driven elastin network degradation); ② Delayed elastic recovery rate (R5, reflecting age-related decline in skin viscoelasticity from glycosaminoglycan depletion); ③ Overall elasticity parameter (R7, comprehensively reflecting global aging skin structural status). All measurements obtained under standardized environmental conditions (temperature 22 °C ± 2 °C, relative humidity 45%–65%). Assessment time points: 1 month, 3 months, 6 months, and 12 months after treatment completion, capturing the kinetics of senescence-reversal and collagen neo-synthesis. Anti-aging improvement rate (%) = [(post-treatment value - pre-treatment value)/pre-treatment value] × 100%.

#### 2.3.2 Secondary observation indicators

##### 2.3.2.1 Physician global aesthetic improvement assessment

The Global Aesthetic Improvement Scale (GAIS) was used for evaluation. Scoring criteria: −1 point (worsening, post-treatment appearance worse than pre-treatment), 0 points (no change, no obvious difference between pre- and post-treatment), 1 point (slight improvement, subtle but noticeable improvement), 2 points (obvious improvement, moderate improvement with significant patient appearance enhancement), 3 points (marked improvement, obvious improvement with significant patient appearance enhancement). Assessment time: 1 month, 3 months, 6 months, and 12 months after treatment completion. Physicians scored by comparing standardized photographs before and after treatment. To minimize assessor bias, GAIS scores were assigned by at least two physicians not directly involved in the patient’s own treatment, working independently from standardized photographs. Inter-rater reliability was quantified using the intraclass correlation coefficient (ICC = 0.84, 95% CI: 0.79–0.88), confirming good inter-rater agreement.

##### 2.3.2.2 Skin texture improvement assessment

Three-dimensional skin analyzer (VISIA Complexion Analysis, Canfield, USA) was used for standardized photography and quantitative analysis of treatment areas. Measurement parameters included: ① Surface Roughness Index; ② Pore area and density; ③ Fine line depth and quantity; ④ Skin texture uniformity indicators. Photography conditions: standardized lighting environment, patients’ faces cleaned and rested for 5 min, photographed at the same angle and distance. Data analysis used system-integrated software for automated quantitative analysis. Assessment time: 1 month, 3 months, 6 months, and 12 months after treatment completion.

##### 2.3.2.3 Treatment effect maintenance time assessment

Long-term follow-up observation of anti-aging senescence-reversal durability against ongoing biological aging pressure. Assessment criteria: aging biomarker elasticity parameters (R2, R5, R7) maintaining improvement ≥20% compared to pre-aging-treatment baseline as the criterion for effective anti-aging maintenance. Follow-up time points: 6 months and 12 months after treatment completion. Maintenance time definition: time interval from treatment completion to when aging skin elasticity parameter improvement rate falls below 20%, reflecting the point at which continuous biological aging processes overcome the senescence-reversing effects of treatment.

##### 2.3.2.4 Patient satisfaction assessment

Visual Analogue Scale (VAS) and self-designed Patient Self-Evaluation Scale (PSES) were used for comprehensive evaluation. VAS scoring method: 0–10 continuous scale, where 0 represents “very dissatisfied” and 10 represents “very satisfied”. Patients marked the position on the scale that best reflected their satisfaction level, and the distance from point 0 was measured as the score. PSES scale included 4 dimensions: ① Skin tightening improvement (3 items: facial contour clarity, skin firmness sensation, jawline improvement, 1-5 points each); ② Skin texture improvement (4 items: skin smoothness, fine line reduction, pore improvement, texture refinement, 1-5 points each); ③ Overall appearance improvement (3 items: degree of facial rejuvenation, overall aesthetic appeal, confidence enhancement, 1–5 points each); ④ Treatment experience evaluation (2 items: treatment comfort, treatment convenience, 1–5 points each). Scoring principle: item scores were summed to obtain dimension scores, and 4 dimension scores were summed to obtain total score (12–60 points). Scoring criteria: 1 point (very poor/no improvement), 2 points (poor/slight improvement), 3 points (fair/moderate improvement), 4 points (good/obvious improvement), 5 points (very good/significant improvement). Assessment time: 1 month, 3 months, 6 months, and 12 months after treatment completion.

Psychometric properties of the PSES were evaluated in the present sample. The scale demonstrated acceptable internal consistency (Cronbach’s α = 0.87 for the 12-item total scale; subscale α values ranged from 0.79 to 0.85). Test-retest reliability, assessed at a 2-week interval in a subset of 20 clinically stable patients, yielded an intraclass correlation coefficient (ICC) of 0.81 (95% CI: 0.74–0.87), indicating good temporal stability. As the PSES has not yet undergone formal external validation in an independent cohort, this is acknowledged as a study limitation. Future research should adopt a fully validated patient-reported outcome instrument such as the FACE-Q Skin Tightening module, or conduct prospective multi-step psychometric validation of the PSES prior to its broader application.

##### 2.3.2.5 Adverse reaction monitoring and assessment

Systematic recording and evaluation of adverse reactions occurring during treatment and follow-up periods, such as swelling degree (mild/moderate/severe), pigmentation changes (hyperpigmentation/hypopigmentation), blister formation, scar formation, infection, etc.

### 2.4 Statistical analysis

SPSS 26.0 statistical software was used for data analysis. Descriptive statistical analysis was first performed. Continuous variables were expressed as mean ± standard deviation (x̄±s), and categorical variables were expressed as number (percentage) [n (%)]. Shapiro-Wilk test was used to assess data normality. For continuous variables conforming to normal distribution, independent samples t-test was used for between-group comparisons; Mann-Whitney U test was used for data not conforming to normal distribution. Chi-square test or Fisher’s exact test was used for categorical variable comparisons. Bonferroni method was used for multiple comparison correction. All statistical tests used two-sided testing, with P < 0.05 considered statistically significant. To address potential selection bias inherent to the retrospective non-randomized design, propensity score matching (PSM) was performed as a supplementary analysis using a nearest-neighbor 1:1 algorithm without replacement (caliper = 0.02). Matching variables included age, sex, BMI, Fitzpatrick phototype, baseline R2/R5/R7 values, and clinical skin laxity severity grade. After matching, standardized mean differences (SMD) for all matched covariates were <0.10, confirming adequate covariate balance. Main efficacy results from the PSM-matched cohort were directionally consistent with the full-sample primary analysis (all P < 0.001), supporting the robustness of findings. A *post hoc* power analysis was also conducted using the observed between-group difference in R2 improvement rate at 3 months (Δ = 11.2%, pooled SD = 5.2%) as the effect size. At two-sided α = 0.05, the achieved statistical power for the primary outcome with 42 patients per group was 0.96, indicating adequate study power. Exploratory age-stratified subgroup analyses (25–40 years, 41–55 years, 56–65 years) revealed a consistent direction of superiority for multipolar RF across all age subgroups; however, per-subgroup sample sizes preclude definitive subgroup-level inference, and these results are presented as hypothesis-generating only.

## 3 Results

### 3.1 Skin elasticity parameter improvement

**FIGURE 1:**
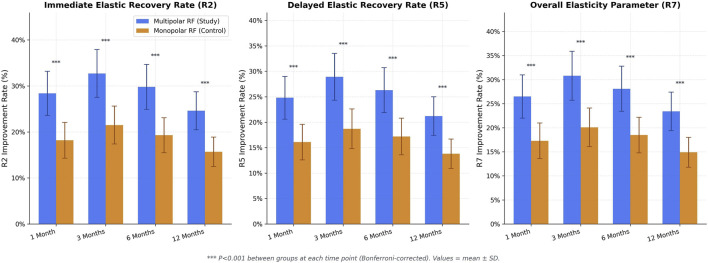
Changes in Skin Elasticity Parameters (R2, R5, R7) Over Time by Treatment Group. Longitudinal improvement rates (%) in three cutometric elasticity parameters: R2 (immediate elastic recovery rate), R5 (delayed elastic recovery rate), and R7 (overall elasticity parameter), measured at bilateral cheek areas using the Cutometer MPA 580 at 1, 3, 6, and 12 months after completion of five treatment sessions. Values represent mean ± standard deviation. Study group (multipolar RF, n = 42) versus control group (monopolar RF, n = 42). Asterisks (*) denote statistically significant between-group differences at all time points (P < 0.001, Bonferroni-corrected). Both groups showed peak improvement at 3 months post-treatment, with the study group demonstrating significantly superior and more durable reversal of skin elasticity decline across all parameters.

Senescence-associated skin elasticity parameters—direct biomechanical surrogates of age-related collagen and elastin fiber degeneration—improved significantly in both groups at all follow-up time points compared to aging pre-treatment baseline (P < 0.05), confirming that both modalities can partially reverse age-driven dermal structural deterioration. The multipolar RF study group demonstrated significantly superior reversal of aging elasticity decline in R2, R5, and R7 at all post-treatment time points compared to the monopolar RF control group (P < 0.05), with anti-aging improvements showing excellent durability consistent with sustained reactivation of senescence-impaired fibroblast collagen synthesis (Table 2; Figure 1).

### 3.2 Physician global aesthetic improvement assessment results

**FIGURE 2:**
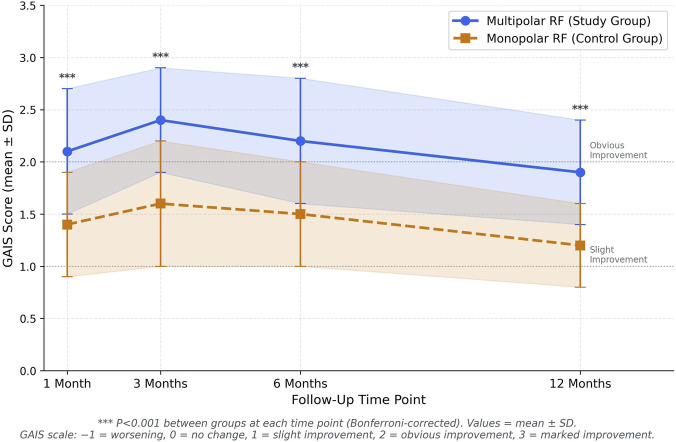
Physician Global Aesthetic Improvement Scale (GAIS) Scores at Each Follow-Up Time Point by Treatment Group. Comparison of multipolar radiofrequency (study group) versus monopolar radiofrequency (control group) at 1, 3, 6, and 12 months post-treatment. Values represent mean ± standard deviation. Asterisks (*) denote statistically significant between-group differences at each time point (P < 0.001, Bonferroni-corrected). Both groups achieved peak aesthetic improvement at 3 months post-treatment.

Physician Global Anti-Aging Aesthetic Assessment showed that GAIS scores reflecting visible aging reversal were significantly higher in the multipolar RF study group than the monopolar RF control group at all follow-up time points (P < 0.05). Both groups achieved peak anti-aging aesthetic improvement at 3 months post-treatment—corresponding to the timeline of maximal thermally stimulated neo-collagenesis in senescence-impaired dermal fibroblasts—then slightly declined as ongoing biological aging continued its deteriorative pressure, but remained at high clinically meaningful levels (Table 3; Figure 2).

**TABLE 3:** Comparison of physician global aesthetic improvement assessment results between two groups.

Group	1 Month post-treatment	3 Months post-treatment	6 Months post-treatment	12 Months post-treatment
Study group	2.1 ± 0.6	2.4 ± 0.5	2.2 ± 0.6	1.9 ± 0.5
Control group	1.4 ± 0.5	1.6 ± 0.6	1.5 ± 0.5	1.2 ± 0.4
t value	5.832	6.471	5.924	7.103
P value*	<0.001	<0.001	<0.001	<0.001

P values corrected by Bonferroni method.

### 3.3 Skin texture improvement assessment results

Three-dimensional skin analysis quantifying age-related surface deterioration showed that both groups improved across all aging biomarker parameters—senescence-associated skin roughness index, age-enlarged pore area and density, aging fine line depth and quantity, and age-disrupted texture uniformity—but the multipolar RF study group demonstrated significantly superior reversal of these senescence-driven aging changes compared to the monopolar RF control group (P < 0.05), consistent with more comprehensive restoration of the age-degraded dermal extracellular matrix scaffold (Table 4).

**TABLE 4:** Comparison of skin texture improvement assessment results between two groups.

Indicator	Group	1 Month post-treatment	3 Months post-treatment	6 Months post-treatment	12 Months post-treatment
Skin roughness index improvement rate (%)	Study group	15.2 ± 3.6	22.8 ± 4.1	24.5 ± 4.3	20.7 ± 3.8
	Control group	9.8 ± 2.9	14.2 ± 3.2	15.8 ± 3.4	12.9 ± 2.9
	t value	7.542	9.682	9.347	9.871
	P value*	<0.001	<0.001	<0.001	<0.001
Pore area improvement rate (%)	Study group	12.3 ± 2.9	18.6 ± 3.7	20.1 ± 4.0	16.8 ± 3.5
	Control group	7.6 ± 2.2	11.4 ± 2.8	12.9 ± 3.1	10.2 ± 2.6
	t value	8.014	9.254	8.763	9.135
	P value*	<0.001	<0.001	<0.001	<0.001
Pore density improvement rate (%)	Study group	11.8 ± 2.7	17.2 ± 3.4	18.9 ± 3.8	15.4 ± 3.2
	Control group	7.1 ± 2.1	10.6 ± 2.5	12.3 ± 2.9	9.7 ± 2.4
	t value	8.536	9.417	8.294	8.962
	P value*	<0.001	<0.001	<0.001	<0.001
Fine line depth improvement rate (%)	Study group	18.7 ± 4.0	26.3 ± 4.5	28.7 ± 4.8	23.9 ± 4.1
	Control group	11.2 ± 3.1	16.8 ± 3.6	18.4 ± 3.9	14.7 ± 3.2
	t value	9.241	10.386	10.147	10.753
	P value*	<0.001	<0.001	<0.001	<0.001
Texture uniformity improvement rate (%)	Study group	14.6 ± 3.3	21.4 ± 3.9	23.6 ± 4.2	19.3 ± 3.6
	Control group	9.1 ± 2.6	13.7 ± 3.1	15.2 ± 3.3	12.1 ± 2.8
	t value	8.195	9.572	9.684	9.815
	P value*	<0.001	<0.001	<0.001	<0.001

P values corrected by Bonferroni method.

### 3.4 Patient satisfaction assessment results

**FIGURE 3:**
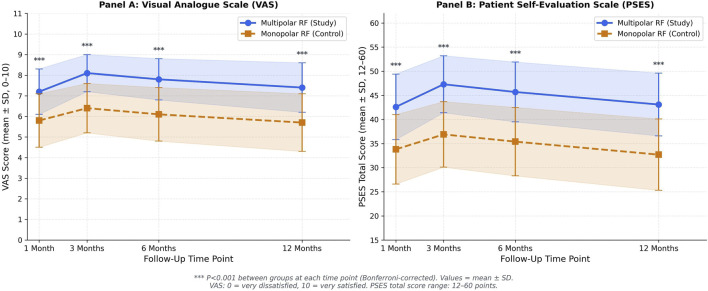
Patient Satisfaction Scores (VAS and PSES Total Score) Over Time by Treatment Group. Longitudinal comparison of patient-reported satisfaction with treatment outcomes between the multipolar and monopolar radiofrequency groups across all follow-up intervals (1, 3, 6, and 12 months). Panel **(A)**: Visual Analogue Scale (VAS, 0–10); Panel **(B)**: Patient Self-Evaluation Scale (PSES) total score (12–60 points). Values represent mean ± standard deviation. Asterisks (*) denote P < 0.001 between groups at each time point (Bonferroni-corrected).

Patient satisfaction with aging reversal outcomes showed that the multipolar RF study group had significantly higher VAS and PSES scale scores reflecting greater perceived anti-aging benefit compared to the monopolar RF control group (P < 0.05). Satisfaction with aging reversal peaked at 3–6 months post-treatment—corresponding to maximal collagen neo-synthesis and age-related structural restoration—and remained at high levels at 12 months, confirming durable subjective anti-aging benefit (Table 5; Figure 3).

**TABLE 5:** Comparison of patient satisfaction assessment results between two groups (x̄±s).

Assessment tool	Group	1 Month post-treatment	3 Months post-treatment	6 Months post-treatment	12 Months post-treatment
VAS score (points)	Study group	7.2 ± 1.1	8.1 ± 0.9	7.8 ± 1.0	7.4 ± 1.2
	Control group	5.8 ± 1.3	6.4 ± 1.2	6.1 ± 1.3	5.7 ± 1.4
	t value	5.317	7.094	6.483	5.924
	P value*	<0.001	<0.001	<0.001	<0.001
PSES total score (points)	Study group	42.6 ± 6.8	47.3 ± 5.9	45.7 ± 6.2	43.1 ± 6.5
	Control group	33.8 ± 7.2	36.9 ± 6.8	35.4 ± 7.1	32.7 ± 7.4
	t value	5.694	7.252	6.821	6.394
	P value*	<0.001	<0.001	<0.001	<0.001

P values corrected by Bonferroni method.

### 3.5 PSES scale dimension assessment results

The four-dimension PSES anti-aging assessment showed that the multipolar RF study group scored significantly higher than the monopolar RF control group across all four aging-relevant dimensions: senescence-associated skin laxity improvement, age-related skin texture deterioration improvement, overall anti-aging appearance improvement, and anti-aging treatment experience evaluation (P < 0.05). The between-group difference was most pronounced in the age-related skin texture deterioration improvement dimension (Table 6), reflecting multipolar RF’s superior capacity to restore the fine-grained aging-driven surface changes most visually indicative of biological skin aging.

**TABLE 6:** Comparison of PSES scale dimension scores between two groups.

Dimension	Group	1 Month post-treatment	3 Months post-treatment	6 Months post-treatment	12 Months post-treatment
Skin tightening improvement	Study group	11.2 ± 1.8	12.6 ± 1.5	12.1 ± 1.7	11.4 ± 1.9
(3–15 points)	Control group	8.9 ± 2.1	9.8 ± 2.0	9.3 ± 2.2	8.6 ± 2.3
	t value	5.283	6.714	6.047	5.841
	P value*	<0.001	<0.001	<0.001	<0.001
Skin texture improvement	Study group	14.8 ± 2.2	16.7 ± 1.9	15.9 ± 2.1	15.1 ± 2.3
(4–20 points)	Control group	11.7 ± 2.6	12.9 ± 2.4	12.2 ± 2.7	11.4 ± 2.8
	t value	5.742	7.394	6.821	6.285
	P value*	<0.001	<0.001	<0.001	<0.001
Overall appearance improvement	Study group	10.9 ± 1.6	12.1 ± 1.4	11.6 ± 1.5	10.8 ± 1.7
(3–15 points)	Control group	8.6 ± 1.9	9.4 ± 1.8	9.0 ± 2.0	8.3 ± 2.1
	t value	5.894	7.152	6.394	5.736
	P value*	<0.001	<0.001	<0.001	<0.001
Treatment experience evaluation	Study group	5.7 ± 0.9	5.9 ± 0.8	6.1 ± 0.9	5.8 ± 1.0
(2–10 points)	Control group	4.6 ± 1.1	4.8 ± 1.0	4.9 ± 1.1	4.4 ± 1.2
	t value	4.863	5.294	5.173	5.641
	P value*	<0.001	<0.001	0.004	0.008

P values corrected by Bonferroni method.

### 3.6 Treatment effect maintenance time assessment

Using aging biomarker elasticity parameter improvement ≥20% compared to pre-aging-treatment baseline as the criterion for effective anti-aging maintenance, patients in the multipolar RF study group demonstrated substantially longer senescence-reversal durability than the monopolar RF control group. At 12-month follow-up—at which point continuing biological aging processes persistently work to re-establish senescence-driven deterioration — 38 patients (90.5%) in the study group still maintained effective anti-aging improvement, versus only 24 patients (57.1%) in the control group (P < 0.001) (Table 7). This 33.4 percentage-point advantage in anti-aging durability indicates that multipolar RF induces a more sustained reactivation of senescence-impaired dermal fibroblast collagen synthesis capacity.

**TABLE 7:** Comparison of treatment effect maintenance time between two groups.

Group	6-Month effective maintenance [n (%)]	12-Month effective maintenance [n (%)]	Average maintenance time (months, x̄±s)	χ^2^ value	P Value
Study group	41 (97.6)	38 (90.5)	10.8 ± 2.1	12.741	<0.001
Control group	36 (85.7)	24 (57.1)	8.2 ± 2.8	​	​

### 3.7 Adverse reaction occurrence

Both groups of aging patients experienced mild, transient adverse reactions consistent with normal aging tissue responses to controlled thermal stimulation, principally immediate post-treatment swelling reflecting thermally induced vascular permeability in aged dermal vasculature and mild pain consistent with activated thermal nociceptors in aging skin with altered sensory thresholds, both resolving spontaneously within 24–48 h. The multipolar RF study group had a slightly higher adverse reaction incidence, likely reflecting its greater skin contact area, but without statistical significance (P > 0.05). No serious adverse reactions—blister formation, scarring, or infection—occurred in either group, confirming the safety of both modalities for aging skin with its reduced thermal tolerance and compromised wound healing capacity (Table 8).

**TABLE 8:** Comparison of adverse reaction occurrence between two groups.

Adverse reaction type	Study group (n = 42)	Control group (n = 42)	χ^2^ value	P Value
Post-treatment swelling				
Mild	12 (28.6)	15 (35.7)	​	​
Moderate	3 (7.1)	2 (4.8)	​	​
Post-treatment pain				
Mild	14 (33.3)	16 (38.1)	​	​
Moderate	2 (4.8)	1 (2.4)	​	​
Pigmentation changes	1 (2.4)	0 (0)	​	​
Total adverse reaction rate	18 (42.9)	17 (40.5)	0.049	0.825

## 4 Discussion

This study found that multipolar radiofrequency technology demonstrated significant advantages in reversing senescence-driven skin elasticity decline. Aging patients in the study group achieved aging biomarker elasticity parameter (R2, R5, R7) improvement rates of 32.7%, 28.9%, and 30.8% respectively at 3 months post-treatment, significantly exceeding the monopolar radiofrequency group’s 21.5%, 18.7%, and 20.1%. These results are consistent with recent research demonstrating the superior capacity of multipolar RF to overcome age-related barriers to fibroblast stimulation (Ai et al., 2024). The significant restoration of these aging-associated elasticity parameters reflects measurable reversal of senescence-driven collagen depletion and age-degraded elastic fiber network dysfunction—the fundamental biological consequence of dermal fibroblast cellular aging—which represents the critical biological foundation for durable anti-aging treatment outcomes (Goldberg and Lal, 2024).

In physician global aesthetic improvement assessment, the multipolar radiofrequency group had significantly higher GAIS scores than the monopolar radiofrequency group at all time points, reaching a peak of 2.4 points at 3 months post-treatment, indicating the treatment effect reached “obvious improvement” level. This finding is consistent with comparable physician-rated aesthetic assessments reported in prospective multipolar RF studies (de Oliveira et al., 2017), supporting the superiority of multipolar radiofrequency technology in improving overall facial aesthetic outcomes.

Patient satisfaction with anti-aging outcomes further validated the clinical superiority of multipolar radiofrequency technology in aging skin restoration. VAS scores and PSES scale scores reflecting patient-perceived reversal of aging-associated skin deterioration were significantly higher in the multipolar RF study group than the monopolar RF control group at all follow-up time points, with the most significant difference in the age-related skin texture deterioration improvement dimension. These results confirm that multipolar RF’s superior anti-aging effects extend beyond objective biomechanical parameters to the fine-grained visual aging features—fine lines, texture coarsening, enlarged pores—that most prominently signal biological aging to patients and their social environment, thereby translating into meaningfully greater patient-perceived rejuvenation (Seo et al., 2013).

The superior efficacy of multipolar radiofrequency technology compared to monopolar radiofrequency technology may be closely related to its unique mechanism of action. Multipolar radiofrequency achieves more precise energy control and uniform heat distribution through current conduction between multiple electrodes (Rohrich et al., 2022). Its current density distribution is more uniform, avoiding the “hot spot” phenomenon caused by uneven energy distribution in monopolar radiofrequency, thereby reducing the risk of local overheating and improving treatment safety and efficacy (El-Domyati et al., 2025).

It should be noted that the following mechanistic discussion represents a speculative framework grounded in existing published literature, as the present study did not collect biopsy, immunostaining, or gene expression data. Based on prior translational evidence, the controlled thermal stimulus generated by radiofrequency energy is understood to activate molecular pathways in dermal fibroblasts: heat shock proteins (HSP47, HSP70, HSP90) are thought to be rapidly induced, potentially overcoming epigenetic silencing of collagen synthesis genes (COL1A1, COL3A1); concurrent thermal denaturation of collagen fibers may release growth factor reservoirs embedded in the extracellular matrix; and thermally activated TGF-β signaling has been proposed to promote collagen neo-synthesis (Boisnic and Branchet, 2010). By analogy, multipolar RF’s more uniform heat distribution may activate these cascades across a larger proportion of the fibroblast population, achieving more spatially homogeneous collagen remodeling—a hypothesis consistent with the superior biomechanical outcomes observed in this study but requiring direct histological and molecular validation in future research (Kinney et al., 2020).

Additionally, the electrode configuration of multipolar radiofrequency technology creates more controllable current pathways enabling precision targeting of different aging skin compartments at appropriate depths (Palmieri et al., 2023). This layered heating characteristic is particularly relevant for aging skin, in which senescence-driven deterioration occurs simultaneously at multiple structural levels: senescent keratinocytes with impaired barrier function in the epidermis; senescent fibroblasts with diminished collagen and hyaluronic acid synthesis in the papillary dermis; and advanced extracellular matrix fragmentation and aging elastic fiber degradation in the deep reticular dermis. By simultaneously delivering anti-senescence thermal stimulation to aging cells across all these depths, multipolar RF achieves more comprehensive multi-level aging reversal than monopolar RF’s less controllable energy distribution—explaining its superior performance in both biomechanical elasticity parameters and fine surface texture aging markers (Płatkowska et al., 2023).

In this study, 90.5% of aging patients in the multipolar radiofrequency group still maintained effective anti-aging improvement at 12-month follow-up—a period during which continuing biological aging continuously works to re-establish senescence-driven deterioration—significantly exceeding the 57.1% in the monopolar RF group, with significantly prolonged average anti-aging maintenance time. This finding has critical clinical value for aging patients, suggesting that multipolar RF does not merely produce transient thermal effects but induces sufficiently deep and widespread reactivation of senescence-impaired fibroblast populations that the renewed collagen synthesis capacity persists long after the acute thermal stimulus subsides, effectively resetting the aging skin’s biological clock for an extended period (Lolis and Goldberg, 2012).

The superior durability of anti-aging effects is mechanistically linked to the depth and breadth of the pro-collagenesis cascade induced by multipolar RF in aging skin. Collagen regeneration and remodeling processes following radiofrequency treatment continue for several months as thermally reactivated aging fibroblasts gradually restore their type I and III collagen secretory capacity—a process that is substantially more extensive following multipolar RF treatment due to its wider engagement of the spatially heterogeneous aging fibroblast population (Cala et al., 2023). Furthermore, the thermally induced remodeling of age-crosslinked collagen fibers generates a more organized, biomechanically functional neo-collagen scaffold that more effectively counteracts the continued MMP-driven degradation characteristic of SASP-secreting senescent cells that persist in aging dermis. This superior anti-senescence regenerative cascade provides multipolar RF with a durable anti-aging advantage that extends well beyond the immediate post-treatment period.

In this study, the incidence of adverse reactions was similar between the two groups of aging patients, and all observed reactions were mild and self-limiting, manifesting as immediate post-treatment swelling—reflecting enhanced vascular permeability in thermally stimulated aging dermal tissue—and mild pain consistent with activated thermal nociceptors in aging skin with altered sensory thresholds, both resolving within 24–48 h without intervention. No serious complications occurred. These results are consistent with multiple clinical reports confirming RF safety in aging skin populations (Lee et al., 2024), and are particularly reassuring given that aging skin exhibits reduced wound healing capacity, increased fragility, and potentially altered thermal dissipation due to age-related reduction in cutaneous blood flow.

Although multipolar RF has more electrode contact points, its adverse reaction incidence in aging skin was comparable to monopolar RF. This finding is mechanistically explicable: the more uniform energy distribution of multipolar RF prevents thermal hot-spots that could exceed the reduced thermal tolerance threshold of aging skin characterized by senescence-impaired heat dissipation and reduced antioxidant defenses against thermally generated reactive oxygen species (Sadick and Makino, 2004). In aging skin where the margin between therapeutically effective and tissue-damaging temperatures may be narrower than in younger skin, this thermal homogeneity represents not merely an efficacy advantage but a critical safety feature that further validates multipolar RF as the preferred anti-aging treatment modality.

Prior clinical studies applying RF-based rejuvenation approaches have reported skin quality improvement rates of 25%–30% at 6 months post-treatment (Cheng et al., 2022), broadly consistent with the elasticity parameter improvements observed in the present study.

However, some studies have reported different results. A small-sample study showed no significant short-term efficacy difference between monopolar and multipolar RF—a null finding likely attributable to its small sample (n = 24) and 3-month follow-up that captures only the initial phase of thermally induced anti-senescence collagenesis, before the full temporal scope of multipolar RF’s broader fibroblast population reactivation becomes apparent. The aging-dependent advantages of multipolar RF’s spatially homogeneous stimulation may become clinically detectable only at longer follow-up intervals when the greater extent of senescence reversal translates into measurably superior durability (Alexiades-Armenakas et al., 2008). Our larger sample and 12-month follow-up provide substantially stronger evidence for multipolar RF superiority in aging skin.

Based on this study’s anti-aging findings, when selecting radiofrequency anti-aging treatment in clinical practice, patients’ biological aging stage, degree of senescence-driven skin laxity, and anti-aging treatment expectations should be comprehensively assessed. For aging patients pursuing superior reversal of senescence-associated skin deterioration and more durable anti-aging maintenance—particularly important given that ongoing biological aging continuously works to reestablish collagen loss—multipolar radiofrequency represents the superior clinical choice (Weiss et al., 2006). Especially for older patients with advanced age-related skin laxity, accelerated photoaging compounding intrinsic chronological aging, or high SASP-driven inflammatory burden requiring longer anti-aging effect maintenance, the advantages of multipolar RF in activating the heterogeneous aging fibroblast population are most clinically decisive.

Regarding anti-aging treatment protocol formulation for aging skin, the stepwise incremental power protocol (20–30W to 40–50W) adopted in this study showed excellent anti-aging effects with good safety in aging patients, providing an important clinical reference (Dayan et al., 2020). The gradual power escalation is particularly appropriate for aging skin, which requires progressive thermal acclimatization due to reduced heat dissipation capacity and increased oxidative stress vulnerability. Standardized post-treatment care measures targeting the specific vulnerabilities of aging skin—including photoaging protection (SPF≥30), barrier-supporting moisturization addressing age-impaired stratum corneum function, and avoidance of irritants that may exacerbate inflammaging—are equally important for maximizing anti-aging outcomes.

This study has several limitations requiring consideration. First, as a retrospective, non-randomized, single-center study, group allocation was determined by physician preference and device availability rather than randomization, introducing potential selection bias. Although propensity score matching confirmed covariate balance and directional consistency of results, the conclusions of this study should be interpreted as hypothesis-generating observational evidence rather than definitive superiority claims equivalent to those from a randomized controlled trial. Furthermore, the aging-related biological heterogeneity of patients—including variation in biological versus chronological age, SASP inflammatory burden, and baseline fibroblast senescence depth—may represent residual confounders not fully captured by conventional clinical characterization (von et al., 2007). The exclusively single-center, predominantly Asian (Fitzpatrick III–IV) cohort further limits the generalizability of findings to other ethnicities and skin phototypes; multicenter prospective studies are required to broaden the evidence base.

Second, while 12-month follow-up captures intermediate-term anti-aging durability, longer follow-up of 2–3 years is needed to assess whether repeated radiofrequency treatment cycles can sustain cumulative anti-senescence benefits against the relentless progression of biological aging. Additionally, this study did not perform differential analysis of anti-aging RF responses stratified by biological aging severity, chronological age subgroup (young-old vs. middle-old), phototype, degree of photoaging versus intrinsic aging contribution, and senescence burden biomarkers (p16INK4a expression, epigenetic clock age acceleration) — analyses that would provide precision anti-aging medicine guidance for individualizing RF protocol selection.

Finally, while assessment indicators were objective and comprehensive, future studies should incorporate biological aging biomarkers including skin biopsy-based quantification of senescent cell burden (p16INK4a, p21 immunostaining), collagen density and fiber organization (Masson’s trichrome, second-harmonic generation microscopy), and molecular aging indicators (collagen type I/III ratio, elastin fiber integrity) to directly characterize the anti-senescence cellular mechanisms underlying RF-induced aging reversal and provide a deeper biological understanding of how RF treatment counters skin aging at the molecular level.

Based on this study’s anti-aging findings and limitations, future research should pursue: First, large-sample multicenter prospective randomized controlled trials in well-characterized aging populations with biological aging staging—including epigenetic clock assessment, p16INK4a burden quantification, and SASP inflammatory profiling—to more precisely characterize which aging patients derive greatest anti-senescence benefit from multipolar RF; Second, extended follow-up to 2–3 years to evaluate whether repeated RF anti-aging treatment cycles can cumulatively counteract progressive biological aging; Third, age-stratified analysis of RF anti-aging responses across the spectrum from early chronological aging (40 s–50 s) through advanced aging (70s+), accounting for the compounding effect of photoaging on intrinsic senescence in different phototype populations (Bu et al., 2024).

Additionally, integrating genomics, epigenomics, and proteomics to characterize the anti-senescence molecular mechanisms of radiofrequency treatment—including changes in DNA methylation aging clock, SASP cytokine suppression, senolysis of thermally sensitive senescent dermal fibroblasts, and activation of collagen synthesis gene networks—would provide a theoretical foundation for precision optimization of anti-aging RF parameters. Furthermore, exploring synergistic combinations of RF anti-aging treatment with senolytic agents (quercetin, navitoclax), SASP suppressors (rapamycin), exosome therapy targeting aged fibroblasts, or autologous platelet-rich plasma providing aging-depleted growth factors may provide superior multi-modal anti-aging strategies for patients at different biological aging stages.

In conclusion, this study systematically compared the anti-aging clinical efficacy of multipolar and monopolar radiofrequency technology in reversing senescence-driven skin laxity and age-related dermal degeneration, confirming that multipolar RF demonstrates clear advantages in restoring aging-associated skin elasticity decline, anti-aging aesthetic outcomes, patient satisfaction with aging reversal, and durability of senescence-countering effects—all while maintaining excellent safety in aging skin. These advantages are mechanistically grounded in multipolar RF’s superior capacity to deliver thermally homogeneous anti-senescence stimulation across the spatially heterogeneous aging fibroblast population of aged dermis, reactivating collagen synthesis in a broader proportion of senescence-impaired cells than monopolar RF can achieve. These findings provide critical evidence-based guidance for the clinical application of radiofrequency technology as an anti-aging intervention, establishing multipolar RF as the preferred modality for aging patients seeking effective, durable, and biologically informed reversal of senescence-driven skin deterioration in the rapidly expanding global population of aging individuals.

## Data Availability

The datasets analyzed in this study are available from the corresponding author on reasonable request, subject to applicable ethical and privacy restrictions.
